# Ca^2+^ in the dorsal raphe nucleus promotes wakefulness *via* endogenous sleep-wake regulating pathway in the rats

**DOI:** 10.1186/s13041-016-0252-0

**Published:** 2016-07-26

**Authors:** Su-Ying Cui, Sheng-Jie Li, Xiang-Yu Cui, Xue-Qiong Zhang, Bin Yu, Yuan-Li Huang, Qing Cao, Ya-Ping Xu, Guang Yang, Hui Ding, Jin-Zhi Song, Hui Ye, Zhao-Fu Sheng, Zi-Jun Wang, Yong-He Zhang

**Affiliations:** Department of pharmacology, Peking University, School of Basic Medical Science, 38 Xueyuan Road, Beijing, 100191 China

**Keywords:** Sleep, Calcium, Dorsal raphe nucleus, Serotonin

## Abstract

Serotonergic neurons in the dorsal raphe nucleus (DRN) are involved in the control of sleep-wake states. Our previous studies have indicated that calcium (Ca^2+^) modulation in the DRN plays an important role in rapid-eye-movement sleep (REMS) and non-REMS (NREMS) regulation during pentobarbital hypnosis. The present study investigated the effects of Ca^2+^ in the DRN on sleep-wake regulation and the related neuronal mechanism in freely moving rats. Our results showed that microinjection of CaCl_2_ (25 or 50 nmol) in the DRN promoted wakefulness and suppressed NREMS including slow wave sleep and REMS in freely moving rats. Application of CaCl_2_ (25 or 50 nmol) in the DRN significantly increased serotonin in the DRN and hypothalamus, and noradrenaline in the locus coeruleus and hypothalamus. Immunohistochemistry study indicated that application of CaCl_2_ (25 or 50 nmol) in the DRN significantly increased c-Fos expression ratio in wake-promoting neurons including serotonergic neurons in the DRN, noradrenergic neurons in the locus coeruleus, and orxinergic neurons in the perifornical nucleus, but decreased c-Fos expression ratio of GABAergic sleep-promoting neurons in the ventrolateral preoptic nucleus. These results suggest that Ca^2+^ in the DRN exert arousal effects *via* up-regulating serotonergic functions in the endogenous sleep-wake regulating pathways.

**Electronic supplementary material:**

The online version of this article (doi:10.1186/s13041-016-0252-0) contains supplementary material, which is available to authorized users.

## Introduction

Dorsal raphe nucleus (DRN) provides the majority of serotonin (5-HT) throughout the central nervous system, including the cerebral cortex, hypothalamus and brain stem [1]. Serotonergic neurons in the DRN play an important role in sleep-wake regulation [2, 3]. Most of the serotonergic neurons in the DRN fire regularly at a slow rate during wakefulness, fire considerably less during non-rapid eye movement sleep (NREMS) and even cease firing during rapid eye movement sleep (REMS) [4, 5]. 5-HT release in many brain regions occurs predominantly during wakefulness, and diminishes at its lowest level during REMS [6]. In the endogenous sleep-wake regulating pathways, the DRN promotes wakefulness *via* excitatory projections to the cerebral cortex and other wakefulness-promoting nuclei, and *via* inhibitory projections to sleep-promoting nuclei [1–3, 7].

Calcium (Ca^2+^) and Ca^2+^ channels express widely throughout the central nervous system and modulate neurotransmitter release and neuron excitability [8, 9]. Numerous in vitro and in vivo studies have supported that the Ca^2+^ current of the serotonergic neurons in the DRN is of prime importance in maintaining 5-HT levels throughout the brain [10–12].

Our previous study indicated that Ca^2+^ modulation in the DRN plays an important role in sleep regulation [13, 14]. We found that up-regulation of Ca^2+^ function in the DRN could reduce NREMS and REMS, but down-regulation of Ca^2+^ function in the DRN could promote NREMS, especially slow wave sleep (SWS) in pentobarbital-treated rats [13]. However the precise mechanism has not been certified yet. The present study investigated the neuroanatomical mechanism of the arousal effects of Ca^2+^ in the DRN. At first, we microinjected CaCl_2_ into the DRN, and monitored sleep-wake behavior in freely moving rats for 6 hours. Then, we measured monoamine neurotransmitters and the neuronal activity in the endogenous sleep-wake regulating brain areas 3 h after CaCl_2_ administration.

## Results

### Effects of CaCl_2_ microinjection in the DRN on sleep parameters

**Fig. 1:**
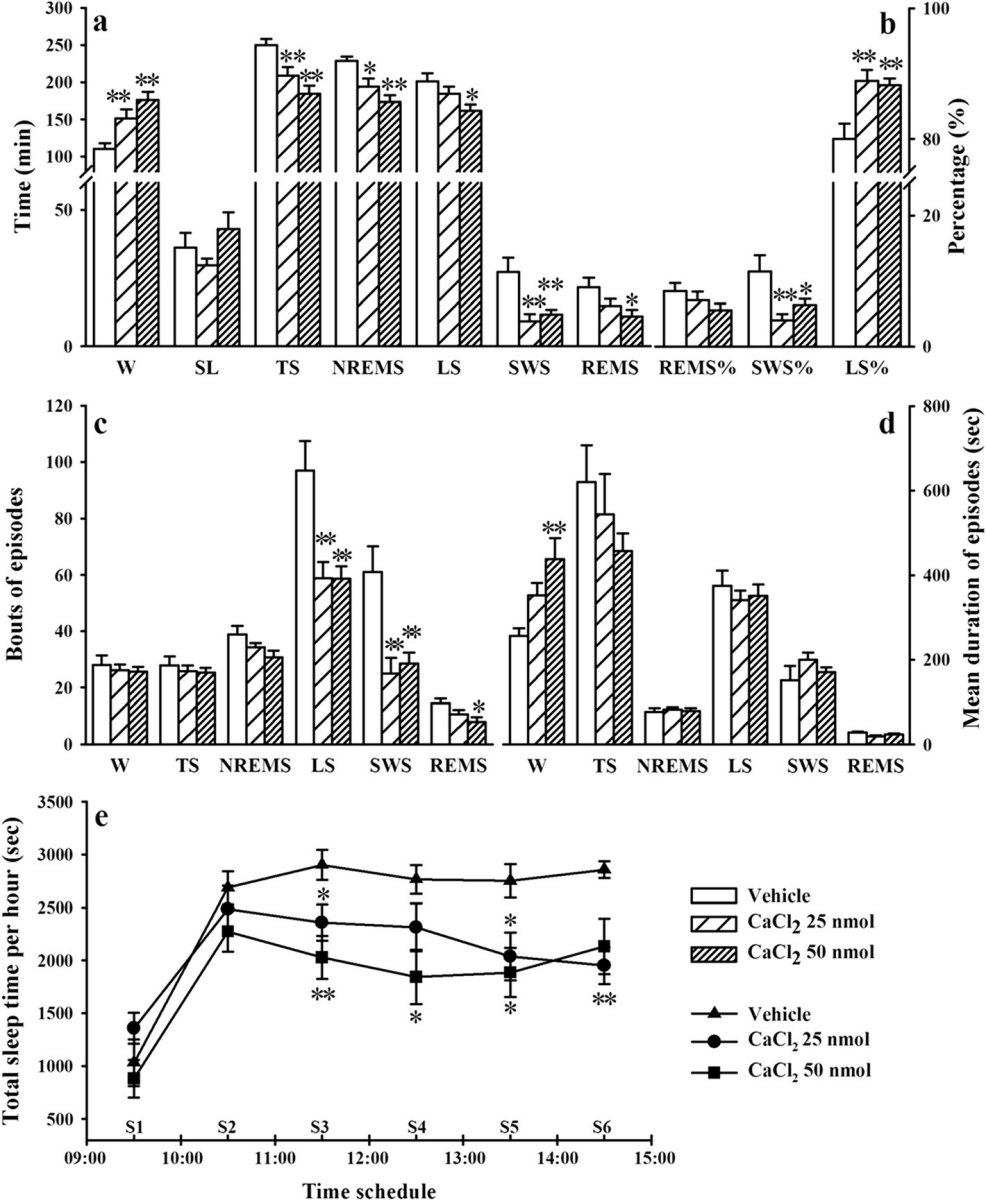
Microinjection of CaCl_2_ (25 or 50 nmol) into the dorsal raphe nucleus (DRN) decreased sleep time in rats. (**a**) Wakefulness (W), total sleep (TS), non-rapid eye movement sleep (NREMS), light sleep (LS), slow wave sleep (SWS) and rapid eye movement sleep (REMS); (**b**) Percentage of LS relative to TS (LS%), percentage of SWS relative to TS (SWS%) and percentage of REMS relative to TS (REMS%); (**c**, **d**) Bouts and mean duration of W, TS, NREMS, LS, SWS and REMS episodes; (**e**) Time spent in TS per 1 h (*n* = 10/group). Data are represented as mean ± SEM, *p < 0.05 and **p < 0.01 *vs* Vehicle (Student-Newman-Keuls test)

Sleep-wake behavior was monitored for 6 h (09:00–15:00) after CaCL_2_ (25 or 50 nmol) was microinjected in the DRN. Microinjection of CaCl_2_ (25 or 50 nmol) in the DRN significantly increased wakefulness (W, *F*_2, 27_ = 10.44, *p* < 0.01, Fig. 1a) and the mean duration of W at dose of 50 nmol (*F*_2, 27_ = 6.75, *p* < 0.01, Fig. 1d). Microinjection of CaCl_2_ (25 or 50 nmol) in the DRN significantly decreased total sleep (TS) time (*F*_2, 27_ = 10.44, *p* < 0.01, Fig. 1a) compared with the vehicle group. Sleep latency (SL) was not influenced by intra-DRN Ca^2+^ administration (Fig. 1a).

The sleep episode analysis revealed that the CaCl_2_ (25 or 50 nmol) microinjection in the DRN significantly reduced NREMS time (*F*_2, 27_ = 9.53, *p* < 0.01, Fig. 1a). Microinjection of CaCl_2_ (25 or 50 nmol) in the DRN decreased light sleep (LS) time (*F*_2, 27_ = 4.34, *p* < 0.05, Fig. 1a) and LS bouts (*F*_2, 27_ = 8.74, *p* < 0.01, Fig. 1c). Microinjection of CaCl_2_ (25 or 50 nmol) in the DRN decreased SWS time (*F*_2, 27_ = 7.55, *p* < 0.01, Fig. 1a) and SWS bouts (*F*_2, 27_ = 9.10, *p* < 0.01, Fig. 1c). Microinjection of the high dose of CaCl_2_ (50 nmol) significantly decreased REMS time (*F*_2, 27_ = 3.67, *p* < 0.05, Fig. 1a) and REMS bouts (*F*_2, 27_ = 4.15, *p* < 0.05, Fig. 1c). Microinjection of CaCl_2_ (25 or 50 nmol) in the DRN significantly increased the percentage of LS relative to TS (LS%, *F*_2, 27_ = 7.57, *p* < 0.01, Fig. 1b) and decreased the percentage of SWS relative to TS (SWS%, *F*_2, 27_ = 5.65, *p* < 0.01, Fig. 1b). The percentage of REMS relative to TS (REMS%) was not influenced by intra-DRN Ca^2+^ administration (Fig. 1b).

TS time was analyzed in 1-h blocks after vehicle or CaCl_2_ (25 or 50 nmol) was microinjected into the DRN. Results indicated TS time was significantly reduced from the 3^rd^ 1-h period (S3) to the 6^th^ 1-h period (S6) in CaCl_2_ groups (25 or 50 nmol) compared with vehicle group (S3, *F*_2, 27_ = 6.41, *p* < 0.01; S4, *F*_2, 27_ = 4.68, *p* < 0.05; S5, *F*_2, 27_ = 4.89, *p* < 0.05; S6, *F*_2, 27_ = 6.62, *p* < 0.01, Fig. 1e).

### Effects of CaCl_2_ microinjection in the DRN on monoamine neurotransmitters

Application of CaCl_2_ in the DRN significantly decreased sleep time. Based on the important effects of serotonergic neurons in the DRN on sleep regulation, we hypothesized that the arousal effects of CaCl_2_ in the DRN might be related to the serotonergic system. Serotonergic projections from the DRN to the prefrontal cortex, hypothalamus and LC are crucial in the sleep-wake cycle [1, 15–17]. Noradrenaline (NE), serotonin (5-HT) and 5-hydroxyindoleacetic acid (5-HIAA, final metabolite of 5-HT) in the DRN, prefrontal cortex, hypothalamus and LC were detected 3 h after intra-DRN CaCl_2_ administration.

**Fig. 2:**
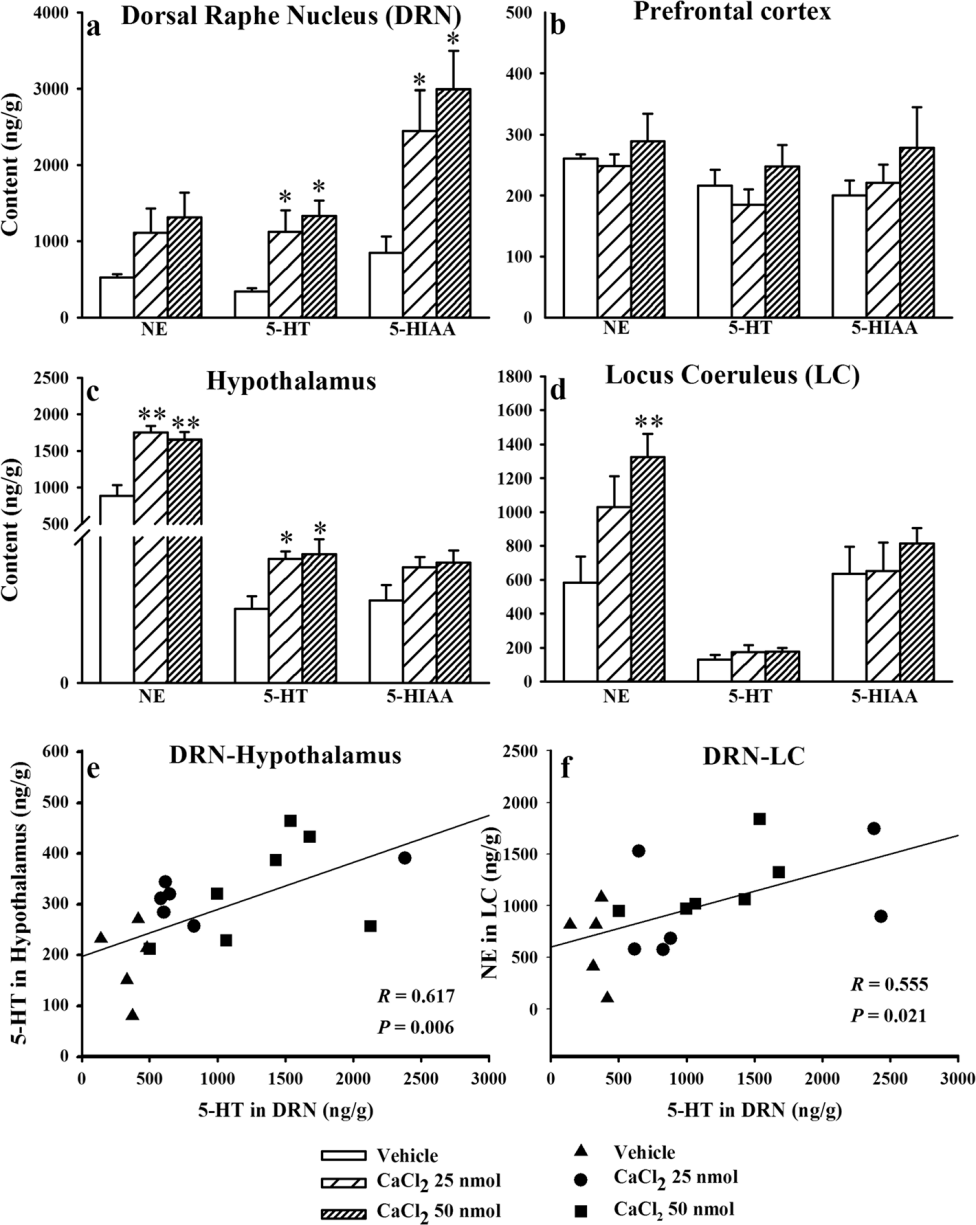
Microinjection of CaCl_2_ (25 or 50 nmol) into the dorsal raphe nucleus (DRN) increased monoamine neurotransmitters. Norepinephrine (NE), serotonin (5-HT) and 5-hydroxyindoleacetic acid (5-HIAA) levels in the dorsal raphe nucleus (**a**), prefrontal cortex (**b**), hypothalamus (**c**), and locus coeruleus (**d**) were analyzed. The relationship between the 5-HT in the DRN and 5-HT in the hypothalamus (**e**) or NE in the LC (**f**) were analyzed (n = 6 ~ 8/group). Data are calculated as ng/g protein and expressed as mean ± SEM, *p < 0.05 and **p < 0.01 *vs* Vehicle (Student-Newman-Keuls test and Pearson’s correlation analysis)

Microinjection of CaCl_2_ (25 or 50 nmol) in the DRN significantly increased 5-HT (*F*_2, 18_ = 5.03, *p* < 0.05) and 5-HIAA (*F*_2, 18_ = 5.09, *p* < 0.05) in the DRN (Fig. 2a), and NE (*F*_2, 15_ = 15.96, *p* < 0.01) and 5-HT (*F*_2, 15_ = 5.17, *p* < 0.05) in the hypothalamus (Fig. 2c). Microinjection of the high dose of CaCl_2_ (50 nmol) significantly increased NE in the LC (*F*_2, 18_ = 5.56, *p* < 0.05, Fig. 2d). Monoamine levels in the prefrontal cortex were not influenced by intra-DRN CaCl_2_ (25 or 50 nmol) administration (Fig. 2b).

The DRN has the highest density of serotonergic neurons in the brain. 5-HT and 5-HIAA in the DRN were increased followed by intra-DRN CaCl_2_ administration. It might be direct effects of Ca^2+^ on serotonergic system. However the effects of intra-DRN CaCl_2_ administration on monoamine levels in the hypothalamus and LC might be occurred secondary to the up-regulating effects of Ca^2+^ on serotonergic neurons. Pearson’s correlation analysis indicated that the level of 5-HT in the DRN was positively correlated with the level of 5-HT in the hypothalamus (*R* = 0.617, *p* < 0.01, Fig. 2e) and the level of NE in the LC (*R* = 0.555, *p* < 0.05, Fig. 2f).

### Effects of CaCl_2_ microinjection in the DRN on neuronal activity in sleep-wake regulating nucleus

Microinjection of CaCl_2_ (25 or 50 nmol) in the DRN suppressed sleep and augmented serotonergic functions in sleep-wake regulating brain areas including DRN, LC and hypothalamus. GABAergic neurons in the ventrolateral preoptic nucleus (VLPO) [18] and histaminergic neurons in the TMN [19] and orexinergic neurons in the perifornical nucleus (Pef) [20] are the most crucial component in the hypothalamic sleep regulation [21]. We performed double-staining immunofluorescence in the DRN, VLPO, TMN, Pef and LC to detect c-Fos expression ratio in specific neurons after 3 h intra-DRN CaCl_2_ application. c-Fos expression is often considered as an index of neuronal activation.

**Fig. 3:**
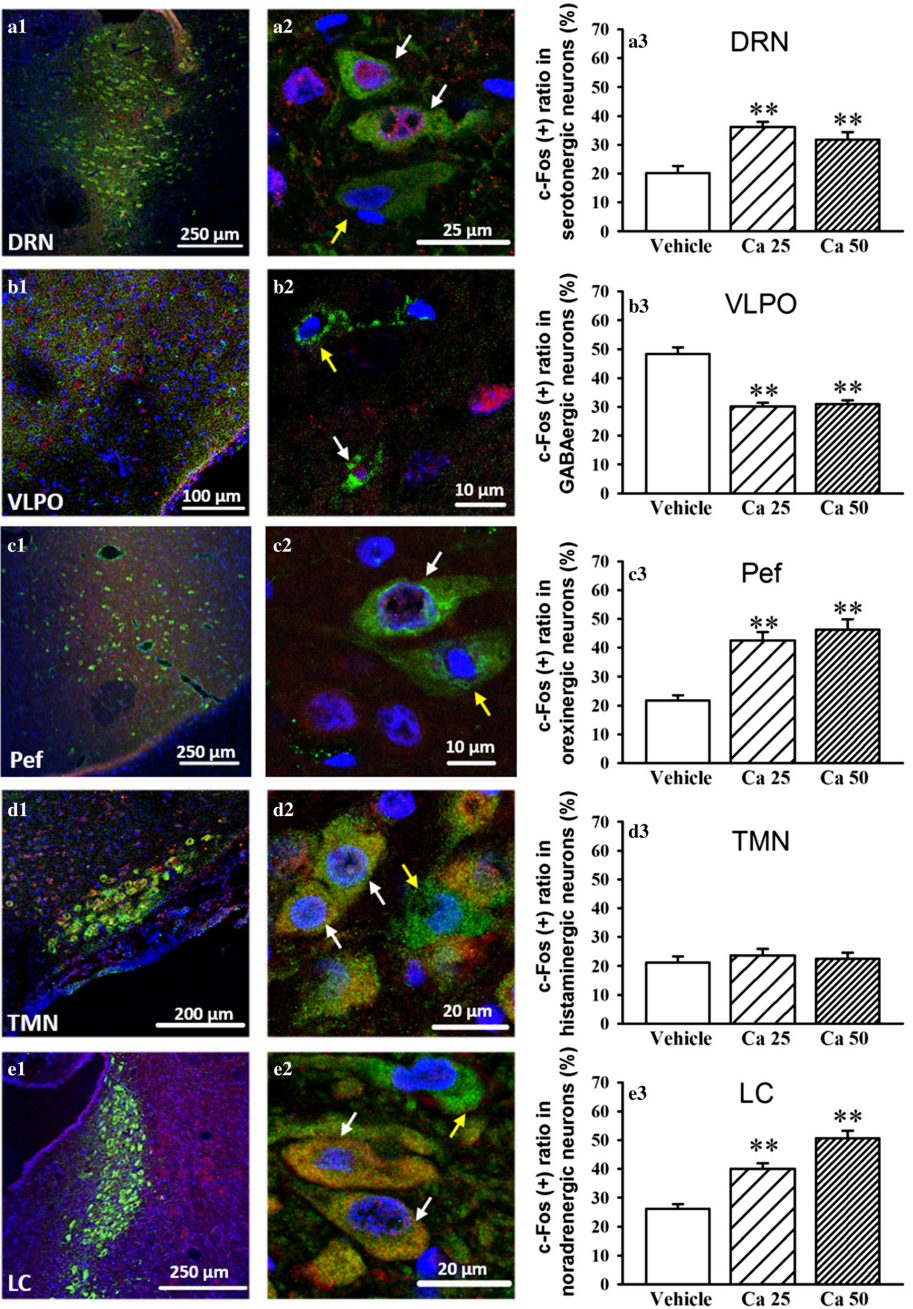
Microinjection of CaCl_2_ (25 or 50 nmol) into the dorsal raphe nucleus (DRN) affected neuronal activity in sleep-wake regulating nucleus. The nucleus-specific neurotransmitter markers were labeled by green, c-Fos was labeled by red and DAPI was labeled by blue. (**a**) In the DRN, nucleus-specific neurotransmitter marker is tryptophan hydroxylase and tryptophan hydroxylase (+) neurons indicate serotonergic neurons. (**b**) In the ventrolateral preoptic nucleus (VLPO), nucleus-specific neurotransmitter marker is glutamic acid decarboxylase and glutamic acid decarboxylase (+) neurons indicate GABAergic neurons. (**c**) In the perifornical nucleus (Pef), nucleus-specific neurotransmitter marker is orexin. (**d**) In tuberomammillary nucleus (TMN), nucleus-specific neurotransmitter marker is adenosine deaminase and adenosine deaminase (+) neurons indicate histaminergic neurons. (**e**) In the locus coeruleus (LC), nucleus-specific neurotransmitter marker is tyrosine hydroxylase and tyrosine hydroxylase (+) neurons indicate noradrenergic neurons. Yellow arrows indicate c-Fos (−)-nucleus-specific neurotransmitter marker (+) neurons. White arrows indicate c-Fos (+)-nucleus-specific neurotransmitter marker (+) neurons. c-Fos (+) ratio in the specific neurons was counted (*n* = 5 ~ 9/group). Data are represented as mean ± SEM, *p < 0.05 and **p < 0.01 *vs* Vehicle (Student-Newman-Keuls test)

Microinjection of CaCl_2_ (25 or 50 nmol) in the DRN significantly increased c-Fos positive ratio of serotonergic neurons in the DRN (*F*_2, 14_ = 13.65, *p* < 0.01, Fig. 3a) and c-Fos positive ratio of orexinergic neurons in the Pef (*F*_2, 14_ = 25.02, *p* < 0.01, Fig. 3c), as well as c-Fos positive ratio of noradrenergic neurons in the LC (*F*_2, 18_ = 34.20, *p* < 0.01, Fig. 3e). Microinjection of CaCl_2_ (25 or 50 nmol) in the DRN significantly decrease c-Fos positive ratio of GABAergic neurons in the VLPO (*F*_2, 24_ = 34.64, *p* < 0.01, Fig. 3b). c-Fos positive ratio of histaminergic neurons in the TMN was not influenced by intra-DRN CaCl_2_ (25 or 50 nmol) administration (Fig. 3d).

## Discussion

CaCl_2_ (25 or 50 nmol) was applied in the DRN at daytime, causing the following principal findings: (*i*) W time significantly increased, and this effect could attribute to increases in mean duration of episodes (Fig. 1), and (*ii*) LS, SWS and REMS significantly decreased, and this effect could attribute to increases in bouts of episodes (Fig. 1), and (*iii*) 5-HT in the DRN and hypothalamus, and NE in the LC and hypothalamus significantly increased (Fig. 2), and (*iv*) c-Fos expression ratio of specific neurons in wake-promoting brain areas (DRN, LC and Pef) significantly increased, but c-Fos expression ratio of GABAergic sleep-promoting neurons in the VLPO significantly decreased (Fig. 3). These results implied that Ca^2+^ in the DRN exerted the arousal effects *via* up-regulating serotonergic functions in endogenous sleep-wake regulating pathway.

Serotonergic neurons in the DRN promote wakefulness and inhibit NREMS and REMS [2, 22]. Serotonergic REM-off neurons in the DRN play suppressive roles in REMS genesis by inhibiting cholinergic REM-on neurons [3, 7]. DRN serotonergic neurons facilitate the consolidation of NREMS by receiving the GABAergic inhibitory inputs from the VLPO, which in turn disinhibits their effects on VLPO [23]. The activity of serotonergic neurons in DRN was modulated by excitatory or inhibitory neurotransmitter and/or neuromodulators from non-serotonergic neurons in the DRN and the synaptic projections derived from all over the brains, which is currently accepted as a crucial component of sleep-wake regulation [2].

Both the Ca^2+^-dependent release of neurotransmitters from the presynaptic membrane and the Ca^2+^-mediating cellular signal transduction in post-synaptic neurons are important mechanisms underneath neurons connection throughout the brain. It has been proved that the orexinergic [8] and glutamatergic [24, 25] exciting signals to the DRN serotonergic neurons were medicated by elevation of Ca^2+^ influx. The research from Barbosa et al. indicates that Ca^2+^ influx is essential for the activation of tryptophan hydroxylase, the rate-limiting enzyme in the 5-HT synthesis, and potentially increases 5-HT release [26]. All of these research provide effective evidence indicating that intra-DRN Ca^2+^ application could potentiate serotonergic system function. The present study shows that intra-DRN Ca^2+^ application increase W and suppress NREMS and REMS (Fig. 1), which is accordance with arousal effects of the DRN serotonergic neurons on sleep-wake regulation [2, 3]. We also detected significant increases in 5-HT and serotonergic neurons activity in the DRN followed by intra-DRN Ca^2+^ application. These results indicate that the arousal effects of Ca^2+^ in the DRN might be related to its up-regulating effects on serotonergic function.

Our previous research indicated that the arousal effects of Ca^2+^ in DRN were associated with activation of protein kinase C (PKC) and calmodulin-dependent kinase II (CaMKII) signaling pathway, since the arousal effects of Ca^2+^ were respectively abolished by PKC inhibitor, chelerythrine chloride, or CaMKII inhibitor, KN-93 [27, 28]. Numerous studies suggest that the Ca^2+^ induced PKC or CaMKII signaling cascade can potentiate the function of the serotonergic system. Based on the present results and previous studies, we hypothesized that the application of Ca^2+^ might potentiate serotonergic function by the activation of PKC and/or CaMKII mediated signal transduction in the DRN. Furthermore, we interestingly noticed that the potential effect of Ca^2+^ on serotonergic function did not only restricted in the DRN, but also stretched to other endogenous sleep-wake regulating pathway.

**Fig. 4:**
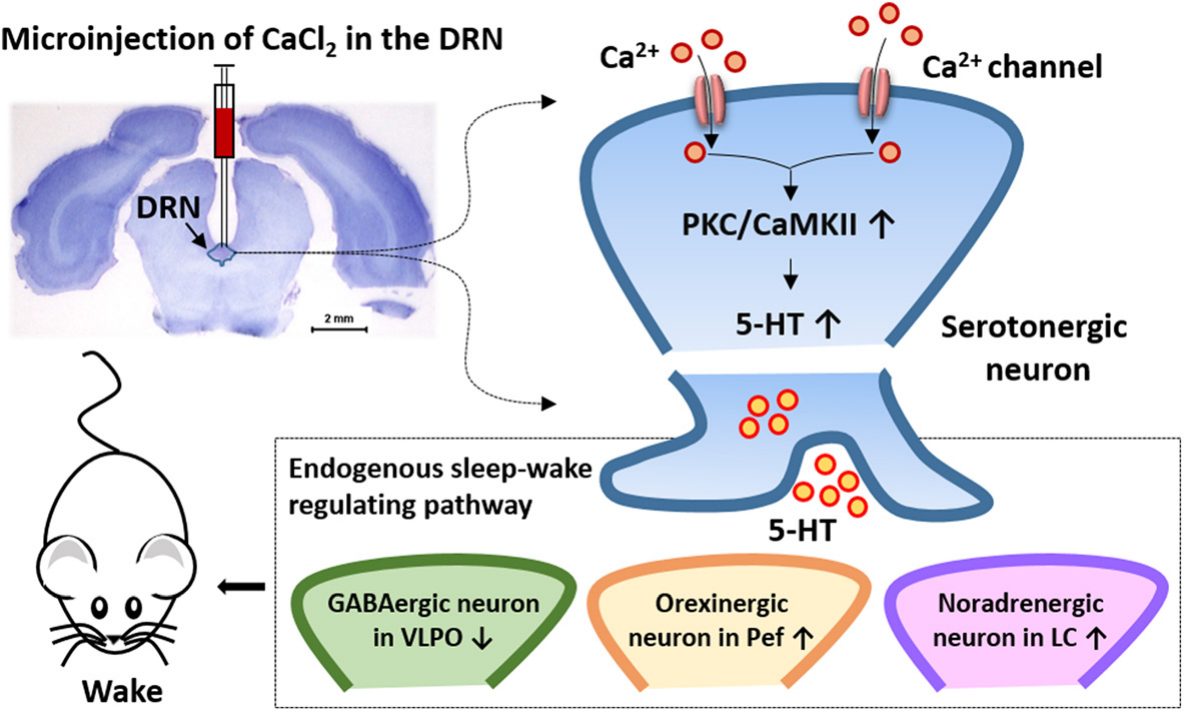
The presumed neuroanatomical mechanism of the arousal effects of Ca^2+^ in the dorsal raphe nucleus (DRN). Microinjection of CaCl_2_ in the DRN promotes wakefulness and suppresses sleep. By activating protein kinase C (PKC) or calmodulin-dependent kinase II (CaMKII) mediated signal transduction, the intra-DRN application of Ca^2+^ might potentiate serotonin (5-HT) synthesis, which up-regulates serotonergic functions in endogenous sleep-wake regulating pathway, causing decreased GABAergic neurons activity in the ventrolateral preoptic nucleus (VLPO), increased orexinergic neurons activity in the perifornical nucleus (Pef) and increased noradrenergic neurons activity in the locus coeruleus (LC)

The sleep-wake regulating pathway in the brain is based on alternating excitation between sleep-promoting neurons and wake-promoting neurons [21, 29]. The sleep-promoting GABAergic neurons in the VLPO project to the wake-promoting neurons including serotonergic neurons in the DRN, noradrenergic neurons in the LC and histaminergic neurons in the TMN, then inhibit their release of neurotransmitters into the cortex and disinhibit their inhibitory effects on the VLPO, which facilitates the consolidation of sleep [21, 29]. The direct mutual inhibition between the VLPO and the monoaminergic cell groups forms a classic flip-flop switch, which produces sharp transitions between sleep and wakefulness. Orexinergic neurons reinforce the arousal systems, which benefit to stabilize the flip-flop switch, like a ‘finger’ on the switch that might prevent unwanted transitions into sleep [21, 29, 30]. The present study shows that the increases in monoamine levels in the hypothalamus and LC followed by intra-DRN Ca^2+^ application were positively correlated with the level of 5-HT in the DRN (Fig. 2). Furthermore, intra-DRN Ca^2+^ application not only significantly increased wake-promoting neurons activity in the DRN, Pef and LC, but also significantly decreased sleep-promoting GABAergic neurons activity in the VLPO (Fig. 3). The changes of neurotransmitters and neuronal activity in endogenous sleep-wake regulating pathway followed by intra-DRN Ca^2+^ application are facilitate to wakefulness, which are accordance with the arousal effects of Ca^2+^ (Fig. 4).

5-HT and the serotonergic neurons activity were increased followed by microinjection of CaCl_2_ in the DRN. It might be direct effects of Ca^2+^ on serotonergic system. However, the changes of monoamine levels and neuronal activity in other endogenous sleep-wake regulating pathway might be occurred secondary to the up-regulating effects of Ca^2+^ on serotonergic system. Serotonergic neural projection from the DRN and serotonergic receptors are detected in the hypothalamus and LC [31–33], which implies the neurons in the hypothalamus and LC might be modulated by 5-HT. Electrical or chemical stimulation of the DRN led to an increased release of 5-HT in the hypothalamus and LC [34, 35] Pharmacological study shows that the agonists of 5HT_1A_ or 5HT_3_ receptor respectively increase noradrenergic activity in the LC [36, 37]. GABAergic sleep-promoting neurons in the VLPO are inhibited by 5-HT and NE [38]. These research provide persuasive evidence supporting the secondary effect of Ca^2+^, since c-Fos expressions in the noradrenergic neurons in the LC were increased and c-Fos expression in the GABAergic neurons in the VLPO were decreased followed by up-regulating effects of Ca^2+^ on serotonergic system. Research from Tabuchi et al. indicated that enhancement of inhibitory serotonergic input to orexinergic neurons *via* 5HT_1A_ receptor caused fragmentation of wakefulness [39]. Present study shows that intra-DRN Ca^2+^ application prolonged mean duration of wakefulness, which might be related to the increase of c-Fos expression in orexinergic neurons. However, it seems that the positive effect on orexinergic neurons induced by Ca^2+^ administration was not medicated by 5HT_1A_ receptor at least, and other serotonergic receptors and neuromodulators should be considerate.

The present study, together with our previous reports [27, 28] demonstrate the application of Ca^2+^ in the DRN promotes wakefulness and suppresses both NREMS and REMS in freely moving rats. By activating PKC or CaMKII mediated signal transduction, the intra-DRN application of Ca^2+^ might potentiate 5-HT synthesis, which up-regulates serotonergic functions in endogenous sleep-wake regulating pathway including DRN, LC, VLPO and Pef (Fig. 4). These findings are critical for our complete understanding of the basic mechanisms of sleep-wake regulation.

## Methods

### Animals

Male Sprague–Dawley rats (220–240 g, Grade I, purchased from the Animal Center of Peking University, Beijing, China) were used. The rats were individually housed in plastic cages and maintained under an artificial 12 h/12 h light/dark cycle (lights on 09:00 to 21:00) at 23 ± 1 °C and 50 ± 10 % humidity. The rats had ad libitum access to food and water. All of the experiments were conducted in accordance with the European Community guidelines for the use of experimental animals and approved by the Peking University Committee on Animal Care and Use.

### Surgery

Surgical procedure details were described previously [40]. The animals were implanted chronically with stainless steel screws over the frontal-parietal cortex and a pair of wire electrodes through the nuchal muscles for recording of electroencephalogram (EEG) and electromyogram (EMG), respectively. Additionally, a guide cannula (26 gauge) was implanted 1 mm above the DRN at coordinates, AP = −8.0; L = 0.0 and DV = −5.8 [41].

### Drugs and drug administration

CaCl_2_ was purchased from Sigma-Aldrich (Louis, MO, USA). CaCl_2_ was dissolved in saline and the pH of the solution was adjusted to 7.3. CaCl_2_ was microinjected into the DRN at 09:00. CaCl_2_ or saline was injected into the rat DRN with an injection cannula (29 gauge), which extended 1 mm beyond the guide, in a 0.2 μl volume over a 2 min period. Histological verification of cannula/injection sites was carried out at the end of the experiments. All the data presented in the present study are derived from animals whose injection site was within the limits of DRN. The location of cannula/injection is shown in Additional file 1: Figure S1 online.

### EEG and EMG recordings and analysis

For the electrophysiological recordings, all of the rats were placed in an electrically shielded box in a noise-attenuated environment with a light-weight shielded cable plugged into the connector on the rat’s head and attached to a counterbalanced swivel. The signals were routed to an electroencephalograph (model MP 150, BIOPAC Systems, CA, USA).

Recordings were performed for 6 h, beginning at 09:00, immediately after CaCl_2_ or vehicle intra-DRN application. The signals were amplified and filtered (EEG, 0.5-30 Hz; EMG, 10–100 Hz) and then digitized at a sampling rate of 128 Hz and recorded using AcqKnowledge software (BIOPAC Systems). The EEG/EMG recordings were analyzed using SleepSign 2.0 software (Kissei, Japan), with the following criteria: W (low-amplitude EEG activity and high-voltage EMG activity), REMS (Fast-fourier transform [FFT] theta ratio of EEG ≥ 60 %, desynchronized EEG, absence of tonic EMG, and occasional body twitches while maintaining a recumbent sleep posture), SWS (FFT delta ratio of EEG ≥ 70 %, large-amplitude, synchronous EEG with sleep spindles present, greatly diminished tonic EMG, eyes closed, small eye movement potentials, and recumbent posture), and LS (FFT delta ratio of EEG < 70 %, high-amplitude slow or spindle EEG activity, and low-amplitude EMG activity). As a final step, the defined sleep-wake stages were examined and corrected according to the visual observation of the animal which was recorded by video camera. NREMS time was equal to SWS time + LS time. TS time was equal to NREMS time + REMS time.

### High-performance liquid chromatography with electrochemical detection

The rats were decapitated 3 h after CaCl_2_ intra-DRN administration (12:00). The DRN, LC, hypothalamus, and prefrontal cortex were dissected and extracted with 0.2 M perchloric acid by ultrasonic homogenation. Details of the neurotransmitter analysis procedure were described previously [42]. High-performance liquid chromatography with electrochemical detection (HPLC-ECD) was used to determine NE, 5-HT and 5-HIAA levels under the following conditions: flow rate (0.60 ml/min), temperature (40 °C), column (Shiseido Capcell Pak C18 MG F90816 column; 3.0 mm inner diameter, 75 mm length, 3 μm pore size), injection volume (20 μl partial loop), mobile phase (0.1 M NaH_2_PO_4_, 0.85 mM OSA, 0.05 mM Na_2_EDTA, 11 % CH_3_OH, pH 3.25 with H_3_PO_4_), detector and conditions (analytical cell: 5011A, E1 = − 175 mV, E2 = + 200 mV; guard cell: 5020, EGC = + 250 mV).

### Immunohistochemistry

The rats were sacrificed 3 h after CaCl_2_ intra-DRN administration (12:00). Under deep anesthesia with chloral hydrate (300 mg/kg, i.p.), the rats were first perfused with 500 ml of 4 % paraformaldehyde. Whole brains were immediately removed and postfixed in the same fixative at 4 °C for 24 h, and then immersed in 30 % sucrose at 4 °C for cryoprotection. The brains were rapidly frozen on liquid *n*-hexane that was cooled with a mixture of solid carbon dioxide and ethanol. Coronal sections that encompassed the VLPO (bregma −0.4 mm ~ −0.8 mm), Pef (bregma −2.8 mm ~ −3.4 mm), TMN (bregma −3.8 mm ~ −4.3 mm), DRN (bregma −7.6 mm ~ −8.3 mm) and LC (bregma −9.7 mm ~ −10.2 mm) [41] were freeze-cut into 20 μm thicknesses with a cryostat (Leica CM1850, Leica Microsystems UK, Milton Keynes, UK).

Each section was immunostained both for nucleus-specific neurotransmitter marker (Additional file 1: Table) and c-Fos. Sections were washed in PBS (3 × 5 min), then incubated in cold acetone for 30 min, followed by washing in PBS (3 × 5 min). Antigen retrieval was conducted in citrate buffer (pH = 6.0) *via* microwave. After sections returned to room temperature naturally, sections were immersed in PBS containing 5 % donkey non-specific serum and 0.3 % Triton X-100 for 30 min. Sections were incubated in the appropriate primary antibodies for specific neurotransmitter markers and c-Fos diluted in PBS containing 1.5 % donkey non-specific serum, 0.3 % Triton X-100 for 12–16 h at 4 °C. After washing in PBS (3 × 5 min), sections were incubated with fluorophore-conjugated donkey anti-rabbit/goat/mouse immunoglobulin G (secondary antibodies) for 120 min at room temperature, washed 3 × 5 min in PBS. At last, the sections were mounted with fluorescent mounting medium with 4′,6-diamidino-2-phenylindole (DAPI). Details of antibodies are summarized in Additional file 1: Table S1 online.

The sections were examined in a confocal microscope (TCS SP8, Leica). Confocal images were processed using Leica LAS AF. The brightness and contrast of captured images were adjusted in Photoshop (Adobe Systems). The nucleus-specific neurotransmitter markers were labeled by green, c-Fos was labeled by red and DAPI was labeled by blue.

In each section, c-Fos positive ratio was counted (the number of c-Fos [+]-nucleus-specific neurotransmitter marker [+] cells divided by the number c-Fos [±]-nucleus-specific neurotransmitter marker [+] cells and then multiply 100 %). Immunoreactive nuclei were counted bilaterally (except for DRN) using at least three serial sections for each area, data were then averaged in order to produce the mean of each group.

### Statistical analysis

The data were analyzed using SPSS 17.0 software and are expressed as mean ± SEM. Multiple comparisons data were analyzed using one-way analysis of variance (ANOVA) followed by the Student-Newman-Keuls *post hoc* test. Pearson’s correlation analysis was performed on pooled data, from both controls and CaCl_2_-treated rats. In all of the tests, *p* < 0.05 was considered statistically significant.

## Abbreviations

5-HIAA, 5-hydroxyindoleacetic acid; 5-HT, serotonin; Ca^2+^, calcium; CaMKII, calmodulin-dependent kinase II; DRN, dorsal raphe nucleus; EEG, electroencephalogram; EMG, electromyogram; FFT, fast Fourier transform; LC, locus coeruleus; LS, light sleep; NE, noradrenaline; NREMS, non-rapid eye movement sleep; Pef, perifornical nucleus; PKC, protein kinase C; REMS, rapid eye movement sleep; SL, Sleep latency; SWS, slow wave sleep; TMN, tuberomammillary nucleus; TS, total sleep; VLPO, ventrolateral preoptic nucleus; W, wakefulness

## Additional file

Additional file 1: Figure S1. Photomicrographs of representative cannula placements in dorsal raphe nucleus (DRN). (a) Sections are according to Paxinos and Watson [41]; (b) Nissle staining in DRN section. **Table S1.** Details of antibody. **Table S2.** Effects of CaCl_2_ microinjection in the DRN on sleep parameters (raw data 1). **Table S3.** Effects of CaCl_2_ microinjection in the DRN on sleep parameters (raw data 2). **Table S4.** Effects of CaCl_2_ microinjection in the DRN on monoamine neurotransmitters (raw data). Table S5. Effects of CaCl_2_ microinjection in the DRN on neuronal activity in sleep-wake regulating nucleus (raw data). (DOCX 413 kb)
