# Supplementary material for: Ca2+ in the dorsal raphe nucleus promotes wakefulness via endogenous sleep-wake regulating pathway in the rats
Source: Mol Brain. 2016 Jul 26;9:71. doi: 10.1186/s13041-016-0252-0 (PMC4960696; doi:10.1186/s13041-016-0252-0)
Supplement: Additional file 1: Figure S1. — Photomicrographs of representative cannula placements in dorsal raphe nucleus (DRN). (a) Sections are according to Paxinos and Watson [41]; (b) Nissle staining in DRN section. Table S1. Details of antibody. Table S2. Effects of CaCl2 microinjection in the DRN on sleep parameters (raw data 1). Table S3. Effects of CaCl2 microinjection in the DRN on sleep parameters (raw data 2). Table S4. Effects of CaCl2 microinjection in the DRN on monoamine neurotransmitters (raw data). Table S5. Effects of CaCl2 microinjection in the DRN on neuronal activity in sleep-wake regulating nucleus (raw data). (DOCX 413 kb) [file 13041_2016_252_MOESM1_ESM.docx]

**Ca^2+^ in the dorsal raphe nucleus promotes wakefulness *via* endogenous sleep-wake regulating pathway in the rats**

Su-Ying Cui^1^, Sheng-Jie Li^1^, Xiang-Yu Cui^1^, Xue-Qiong Zhang^1^, Bin Yu^1^, Yuan-Li Huang^1^, Qing Cao^1^, Ya-Ping Xu^1^, Guang Yang^1^, Hui Ding^1^, Jin-Zhi Song^1^, Hui Ye^1^, Zhao-Fu Sheng^1^, Zi-Jun Wang^1^ & Yong-He Zhang^1*^

^1^ Department of pharmacology, Peking University, School of Basic Medical Science, 38 Xueyuan Road, Beijing, 100191, China.

^*^Correspondence: [zhyh@hsc.pku.edu.cn](mailto:zhyh@hsc.pku.edu.cn)

| Su-Ying Cui: [csy@bjmu.edu.cn](mailto:csy@bjmu.edu.cn) | Sheng-Jie Li: [lsj890721@163.com](mailto:lsj890721@163.com) |
| --- | --- |
| Xiang-Yu Cui: [cxy88@126.com](mailto:cxy88@126.com) | Xue-Qiong Zhang: [xqzh89@163.com](mailto:xqzh89@163.com) |
| Bin Yu: [yubin@bjmu.edu.cn](mailto:yubin@bjmu.edu.cn) | Yuan-Li Huang: [hyli0309@126.com](mailto:hyli0309@126.com) |
| Qing Cao: [caoqing_2503@163.com](mailto:caoqing_2503@163.com) | Ya-Ping Xu: [xyp19890905@sina.com](mailto:xyp19890905@sina.com) |
| Guang Yang: [yangguangfly@126.com](mailto:yangguangfly@126.com) | Hui Ding: [dinghui_dh88@sina.com](mailto:dinghui_dh88@sina.com) |
| Jin-Zhi Song: [sjzhinx@163.com](mailto:sjzhinx@163.com) | Hui Ye: [kimirafael@126.com](mailto:kimirafael@126.com) |
| Zhao-Fu Sheng: [jianbingxuan@163.com](mailto:jianbingxuan@163.com) | Zi-Jun Wang: [zijunwan@buffalo.edu](mailto:zijunwan@buffalo.edu) |


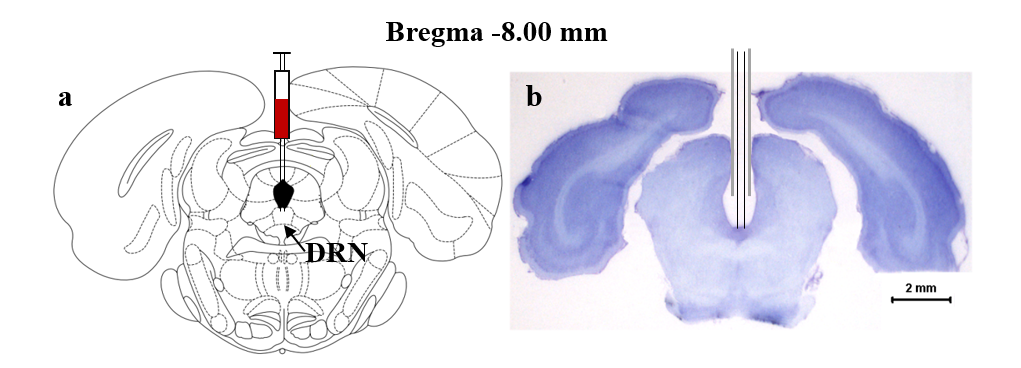


**Figure S1.** Photomicrographs of representative cannula placements in dorsal raphe nucleus (DRN). (a) Sections are according to Paxinos and Watson^1^; (b) Nissle staining in DRN section.

1. Paxinos G, Watson C. The Rat Brain in Stereotaxic Coordinates. 4th ed. San Diego: Academic Press; 1986.

**Table S1. Details of antibody**

| **Brain area (Specific neurotransmitter)** | **Neurotransmitter markers** | **Primary antibodies** | **Secondary antibodies** |
| --- | --- | --- | --- |
| VLPO  (GABA) | GAD | Mouse anti-GAD antibody (Millipore, MAB-5406, 1:500)  Rabbit anti-c-Fos antibody (Santa Cruz, sc-52, 1:200) | Green Donkey-Anti-Mouse IgG (EarthOx, E032211, 1:500)  Red Donkey Anti-Rabbit IgG (IFKine, A24421, 1:500) |
| Pef  (Orexin) | Orexin | Goat anti-Orexin antibody (Santa Cruz, sc-8070, 1:100)  Rabbit anti-c-Fos antibody (Santa Cruz, sc-52, 1:200) | Green Donkey-Anti-Goat IgG (Thermo, A-11055, 1:500)  Red Donkey Anti-Rabbit IgG (IFKine, A24421, 1:500) |
| TMN  (Histamine) | ADA | Rabbit anti-ADA antibody (Millipore, AB176, 1:800)  Goat anti-c-Fos antibody (Abcam, ab87655, 1:400) | Green Donkey Anti-Rabbit IgG (IFKine, A24221, 1:500)  Red Donkey Anti-Goat IgG (Thermo, A-11058, 1:500) |
| DRN  (5-HT) | TrpOH | Sheep anti-TrpOH antibody (AbD, 9260-2505, 1:2000)  Rabbit anti-c-Fos antibody (Santa Cruz, sc-52, 1:200) | Green Donkey-Anti-Sheep IgG (Abcam, ab150177, 1:500)  Red Donkey Anti-Rabbit IgG (IFKine, A24421, 1:500) |
| LC  (NE) | TH | Rabbit anti-TH antibody (Santa Cruz, sc-14007, 1:2000)  Goat anti-c-Fos antibody (Abcam, ab87655, 1:400) | Green Donkey Anti-Rabbit IgG (IFKine, A24221, 1:500)  Red Donkey Anti-Goat IgG (Thermo, A-11058, 1:500) |

5-HT: serotonin; ADA: adenosine deaminase; DRN: dorsal raphe nucleus; GAD: glutamic acid decarboxylase; GABA: γ-aminobutyric acid; LC: locus coeruleus; NE: norepinephrine; Pef: perifornical nucleus; TH: tyrosine hydroxylase; TMN: tuberomammillary nucleus; TrpOH: tryptophan hydroxylase; VLPO: ventrolateral preoptic nucleus.

**Table S2. Effects of CaCl_2_ microinjection in the DRN on sleep parameters (raw data 1).**

| **Group** | **No.** | **Time (min)** | | | | | | | **Percentage (%)** | | | **Bouts of episodes** | | | | | **Mean duration of episodes (sec)** | | | | |
| --- | --- | --- | --- | --- | --- | --- | --- | --- | --- | --- | --- | --- | --- | --- | --- | --- | --- | --- | --- | --- | --- |
|  |  | **W** | **SL** | **TS** | **NREMS** | **LS** | **SWS** | **REMS** | **REMS%** | **SWS%** | **LS%** | **W** | **TS** | **NREMS** | **LS** | **SWS** | **W** | **TS** | **NREMS** | **LS** | **SWS** |
| **Vehicle** | 1 | 108.5 | 52.7 | 251.7 | 238.8 | 206.7 | 32.2 | 12.8 | 5.10 | 12.78 | 82.12 | 37 | 37 | 52 | 156 | 110 | 15 | 175 | 407 | 275 | 79 |
|  | 2 | 134.2 | 24.7 | 226.0 | 210.3 | 184.0 | 26.3 | 15.7 | 6.93 | 11.65 | 81.42 | 40 | 40 | 48 | 97 | 49 | 10 | 201 | 338 | 262 | 113 |
|  | 3 | 82.2 | 20.3 | 278.0 | 245.3 | 238.8 | 6.5 | 32.7 | 11.75 | 2.34 | 85.91 | 24 | 24 | 28 | 97 | 69 | 7 | 260 | 640 | 528 | 131 |
|  | 4 | 83.7 | 15.3 | 276.5 | 250.7 | 246.7 | 4.0 | 25.8 | 9.34 | 1.45 | 89.21 | 17 | 17 | 27 | 36 | 10 | 13 | 295 | 975 | 556 | 410 |
|  | 5 | 92.5 | 27.2 | 267.7 | 226.5 | 193.0 | 33.5 | 41.2 | 15.38 | 12.52 | 72.10 | 16 | 16 | 35 | 99 | 65 | 22 | 346 | 1003 | 388 | 116 |
|  | 6 | 82.8 | 38.2 | 277.3 | 249.3 | 242.5 | 6.8 | 28.0 | 10.10 | 2.46 | 87.44 | 17 | 17 | 34 | 55 | 21 | 18 | 292 | 978 | 439 | 264 |
|  | 7 | 155.7 | 63.0 | 204.5 | 189.8 | 137.8 | 52.0 | 14.7 | 7.17 | 25.43 | 67.40 | 46 | 45 | 52 | 125 | 85 | 15 | 203 | 272 | 218 | 66 |
|  | 8 | 114.5 | 28.7 | 245.7 | 218.8 | 174.3 | 44.5 | 26.8 | 10.92 | 18.11 | 70.96 | 32 | 31 | 44 | 114 | 73 | 22 | 214 | 475 | 298 | 91 |
|  | 9 | 129.3 | 28.8 | 230.8 | 225.2 | 185.5 | 39.7 | 5.7 | 2.45 | 17.18 | 80.36 | 27 | 27 | 34 | 102 | 71 | 8 | 287 | 512 | 397 | 109 |
|  | 10 | 117.5 | 62.2 | 242.7 | 229.3 | 202.7 | 26.7 | 13.3 | 5.49 | 10.99 | 83.52 | 24 | 24 | 35 | 88 | 57 | 15 | 293 | 606 | 392 | 138 |
| **CaCl_2_ 25 nmol** | 1 | 165.3 | 37.2 | 194.8 | 189.7 | 185.3 | 4.3 | 5.2 | 2.65 | 2.22 | 95.12 | 34 | 34 | 38 | 59 | 21 | 4 | 291 | 343 | 299 | 188 |
|  | 2 | 175.8 | 29.7 | 184.3 | 177.3 | 168.2 | 9.2 | 7.0 | 3.80 | 4.97 | 91.23 | 31 | 31 | 35 | 70 | 35 | 5 | 340 | 356 | 303 | 144 |
|  | 3 | 123.2 | 29.0 | 237.0 | 225.3 | 206.7 | 18.7 | 11.7 | 4.92 | 7.88 | 87.20 | 28 | 28 | 35 | 94 | 61 | 8 | 263 | 507 | 386 | 131 |
|  | 4 | 61.5 | 22.2 | 298.7 | 275.3 | 247.5 | 27.8 | 23.3 | 7.81 | 9.32 | 82.87 | 14 | 14 | 32 | 75 | 43 | 19 | 263 | 1279 | 515 | 197 |
|  | 5 | 169.7 | 30.0 | 190.5 | 180.7 | 178.5 | 2.2 | 9.8 | 5.16 | 1.14 | 93.70 | 23 | 22 | 29 | 40 | 11 | 8 | 442 | 519 | 373 | 267 |
|  | 6 | 146.0 | 39.5 | 214.2 | 196.7 | 193.3 | 3.3 | 17.5 | 8.17 | 1.56 | 90.27 | 26 | 26 | 37 | 52 | 15 | 12 | 336 | 493 | 318 | 222 |
|  | 7 | 173.5 | 28.5 | 186.7 | 154.2 | 144.0 | 10.2 | 32.5 | 17.41 | 5.45 | 77.14 | 21 | 20 | 33 | 58 | 26 | 16 | 495 | 560 | 280 | 148 |
|  | 8 | 166.5 | 20.3 | 193.7 | 178.8 | 177.8 | 1.0 | 14.8 | 7.66 | 0.52 | 91.82 | 28 | 28 | 34 | 37 | 3 | 10 | 356 | 414 | 315 | 288 |
|  | 9 | 195.5 | 40.0 | 164.7 | 152.8 | 149.3 | 3.5 | 11.8 | 7.19 | 2.13 | 90.69 | 24 | 23 | 27 | 38 | 11 | 8 | 488 | 429 | 339 | 235 |
|  | 10 | 140.5 | 20.7 | 219.7 | 206.0 | 195.0 | 11.0 | 13.7 | 6.22 | 5.01 | 88.77 | 33 | 33 | 43 | 66 | 24 | 16 | 255 | 399 | 287 | 177 |
| **CaCl_2_ 50 nmol** | 1 | 237.7 | 59.2 | 122.5 | 117.3 | 110.2 | 7.2 | 5.2 | 4.22 | 5.85 | 89.93 | 24 | 23 | 26 | 49 | 23 | 3 | 593 | 319 | 270 | 134 |
|  | 2 | 181.7 | 45.8 | 178.5 | 171.0 | 162.3 | 8.7 | 7.5 | 4.20 | 4.86 | 90.94 | 22 | 22 | 29 | 48 | 19 | 8 | 495 | 486 | 353 | 202 |
|  | 3 | 150.7 | 24.8 | 209.5 | 198.0 | 178.7 | 19.3 | 11.5 | 5.49 | 9.23 | 85.28 | 32 | 31 | 40 | 68 | 29 | 12 | 282 | 405 | 297 | 157 |
|  | 4 | 150.7 | 35.0 | 209.5 | 187.2 | 179.3 | 7.8 | 22.3 | 10.66 | 3.74 | 85.60 | 34 | 34 | 43 | 59 | 17 | 17 | 265 | 369 | 261 | 182 |
|  | 5 | 132.5 | 45.3 | 227.7 | 203.7 | 187.8 | 15.8 | 24.0 | 10.54 | 6.95 | 82.50 | 27 | 26 | 36 | 86 | 50 | 14 | 294 | 525 | 339 | 131 |
|  | 6 | 200.0 | 17.7 | 160.2 | 159.5 | 138.0 | 21.5 | 0.7 | 0.42 | 13.42 | 86.16 | 27 | 27 | 28 | 67 | 42 | 1 | 444 | 355 | 341 | 123 |
|  | 7 | 208.7 | 40.2 | 151.5 | 148.0 | 142.3 | 5.7 | 3.5 | 2.31 | 3.74 | 93.95 | 30 | 30 | 30 | 46 | 17 | 3 | 417 | 302 | 295 | 185 |
|  | 8 | 196.5 | 25.7 | 163.7 | 156.3 | 149.3 | 7.0 | 7.3 | 4.48 | 4.28 | 91.24 | 15 | 14 | 17 | 37 | 20 | 5 | 785 | 701 | 551 | 242 |
|  | 9 | 148.8 | 86.8 | 211.3 | 200.2 | 187.5 | 12.7 | 11.2 | 5.28 | 5.99 | 88.72 | 21 | 21 | 28 | 69 | 42 | 7 | 425 | 603 | 428 | 162 |
|  | 10 | 153.2 | 48.3 | 207.0 | 191.5 | 181.2 | 10.3 | 15.5 | 7.49 | 4.99 | 87.52 | 24 | 24 | 30 | 57 | 27 | 9 | 382 | 517 | 383 | 190 |

LS: light sleep; LS%: LS%, percentage of LS relative to TS; NREMS: non-rapid eye movement sleep; REMS: rapid eye movement sleep; REMS%, percentage of REMS relative to TS; SWS: slow wave sleep; SWS%, percentage of SWS relative to TS; TS: total sleep; W: wakefulness.

**Table S3. Effects of CaCl_2_ microinjection in the DRN on sleep parameters (raw data 2).**

| **Group** | **No.** | **Total sleep time (sec)** | | | | | |
| --- | --- | --- | --- | --- | --- | --- | --- |
|  |  | **S1** | **S2** | **S3** | **S4** | **S5** | **S6** |
| **Vehicle** | 1 | 440 | 3060 | 2430 | 2980 | 3020 | 3160 |
|  | 2 | 790 | 2500 | 2490 | 2430 | 2440 | 2900 |
|  | 3 | 1940 | 3430 | 3070 | 2730 | 2350 | 3150 |
|  | 4 | 1600 | 2080 | 3450 | 3240 | 3350 | 2860 |
|  | 5 | 1720 | 2910 | 2100 | 2920 | 3490 | 2910 |
|  | 6 | 1050 | 2570 | 3460 | 3530 | 2950 | 3070 |
|  | 7 | 0 | 2340 | 3210 | 2130 | 2030 | 2550 |
|  | 8 | 1370 | 2590 | 3050 | 2850 | 2100 | 2780 |
|  | 9 | 1410 | 2010 | 2890 | 2330 | 2800 | 2400 |
|  | 10 | 0 | 3380 | 2870 | 2520 | 2990 | 2790 |
| **CaCl_2_ 25 nmol** | 1 | 1370 | 3210 | 1710 | 1580 | 1940 | 1870 |
|  | 2 | 1470 | 2140 | 2690 | 2180 | 1030 | 1540 |
|  | 3 | 1610 | 2500 | 1990 | 2180 | 2890 | 3040 |
|  | 4 | 2110 | 3420 | 3390 | 3370 | 2950 | 2670 |
|  | 5 | 840 | 3240 | 2280 | 1110 | 2340 | 1620 |
|  | 6 | 1230 | 2990 | 1720 | 1990 | 2570 | 2340 |
|  | 7 | 1780 | 1340 | 2790 | 1950 | 1900 | 1440 |
|  | 8 | 1430 | 1920 | 2260 | 2870 | 1620 | 1510 |
|  | 9 | 430 | 2140 | 1960 | 2800 | 860 | 1690 |
|  | 10 | 1310 | 1940 | 2780 | 3110 | 2260 | 1770 |
| **CaCl_2_ 50 nmol** | 1 | 50 | 2980 | 1200 | 920 | 1270 | 930 |
|  | 2 | 850 | 1230 | 950 | 2310 | 2870 | 2490 |
|  | 3 | 930 | 2160 | 3130 | 2120 | 2310 | 1920 |
|  | 4 | 1350 | 2910 | 1840 | 2100 | 2170 | 2200 |
|  | 5 | 880 | 2830 | 2290 | 2360 | 2880 | 2420 |
|  | 6 | 1900 | 2570 | 1760 | 500 | 1170 | 1700 |
|  | 7 | 890 | 1640 | 1950 | 800 | 1490 | 2310 |
|  | 8 | 1250 | 2690 | 2560 | 1800 | 680 | 840 |
|  | 9 | 0 | 1800 | 2450 | 2670 | 2330 | 3420 |
|  | 10 | 700 | 1930 | 2160 | 2850 | 1690 | 3090 |

Time spent in total sleep per 1 h. S1: 21:00~22:00; S2: 22:00~23:00; S3: 23:00~24:00; S4: 00:00~01:00; S5: 01:00~02:00; S6: 02:00~03:00.

**Table S4. Effects of CaCl_2_ microinjection in the DRN on monoamine neurotransmitters (raw data).**

| **Group** | **No.** | **Dorsal raphe nucleus (ng/g)** | | | **Prefrontal cortex (ng/g)** | | | **Hypothalamus (ng/g)** | | | **Locus coeruleus (ng/g)** | | |
| --- | --- | --- | --- | --- | --- | --- | --- | --- | --- | --- | --- | --- | --- |
|  |  | **NE** | **5-HA** | **5-HIAA** | **NE** | **5-HA** | **5-HIAA** | **NE** | **5-HA** | **5-HIAA** | **NE** | **5-HA** | **5-HIAA** |
| **Vehicle** | 1 |  |  |  | 263.19 | 272.00 | 166.84 |  |  |  | 265.23 | 210.51 | 1033.01 |
|  | 2 | 495.09 | 416.07 | 640.86 | 261.17 | 314.91 | 317.49 | 660.97 | 268.08 | 232.30 | 103.14 | 52.37 | 57.17 |
|  | 3 | 556.98 | 312.05 | 640.86 | 245.03 | 179.06 | 160.06 |  |  |  | 412.58 | 124.37 | 857.95 |
|  | 4 | 660.12 | 372.73 | 461.42 | 297.63 | 272.55 | 269.10 | 656.53 | 78.17 | 70.69 | 1079.98 | 203.95 | 953.21 |
|  | 5 | 323.18 | 141.58 | 311.89 | 259.29 | 174.57 | 173.57 | 1109.00 | 229.53 | 265.57 | 814.84 | 65.01 | 280.96 |
|  | 6 | 543.85 | 480.68 | 1444.85 | 260.02 | 124.31 | 164.03 | 1351.41 | 211.12 | 296.22 |  |  |  |
|  | 7 | 584.48 | 332.28 | 1580.79 | 237.93 | 174.96 | 149.40 | 621.29 | 148.89 | 180.95 | 817.51 | 118.78 | 624.37 |
| **CaCl_2_ 25 nmol** | 1 | 1005.65 | 585.09 | 1105.49 | 327.16 | 247.18 | 165.22 | 1770.75 | 308.69 | 217.07 |  |  |  |
|  | 2 | 913.56 | 829.66 | 2471.89 | 266.61 | 207.83 | 128.92 | 1693.92 | 254.10 | 235.10 | 571.26 | 206.70 | 1507.86 |
|  | 3 | 1196.47 | 884.14 | 1435.53 | 267.54 | 215.37 | 187.23 |  |  |  | 680.75 | 303.38 | 855.96 |
|  | 4 | 1186.15 | 2434.28 | 3492.69 | 125.63 | 66.15 | 178.28 |  |  |  | 893.91 | 16.05 | 203.28 |
|  | 5 | 353.64 | 619.15 | 3771.93 | 261.02 | 273.13 | 254.25 | 1344.00 | 341.46 | 265.74 | 577.61 | 108.35 | 300.57 |
|  | 6 | 3197.46 | 2383.72 | 5062.80 | 288.62 | 118.17 | 405.78 | 1905.35 | 388.49 | 365.87 | 1744.07 | 315.20 | 754.39 |
|  | 7 |  |  |  | 226.48 | 164.27 | 276.96 |  |  |  | 1222.99 | 179.55 | 429.38 |
|  | 8 | 550.10 | 650.10 | 918.57 | 190.91 | 109.36 | 131.95 | 1891.57 | 317.70 | 368.63 | 1526.53 | 95.35 | 505.30 |
|  | 9 | 489.93 | 606.76 | 1329.79 | 278.88 | 263.36 | 261.70 | 1896.20 | 281.89 | 307.61 |  |  |  |
| **CaCl_2_ 50 nmol** | 1 | 1974.47 | 1064.93 | 1904.27 |  |  |  | 2058.16 | 225.87 | 323.73 | 1018.55 | 157.11 | 596.23 |
|  | 2 | 268.17 | 502.75 | 1922.58 | 234.42 | 236.96 | 128.17 | 1455.31 | 209.36 | 159.68 | 948.92 | 231.15 | 757.95 |
|  | 3 | 1402.76 | 996.83 | 1871.32 | 264.59 | 309.98 | 253.56 | 1323.37 | 318.46 | 247.11 | 972.50 | 206.38 | 877.43 |
|  | 4 |  |  |  | 577.61 | 412.77 | 729.97 |  |  |  | 1746.57 | 86.68 | 1367.80 |
|  | 5 |  |  |  | 160.70 | 134.40 | 264.99 |  |  |  | 1688.98 | 167.94 | 506.39 |
|  | 6 | 2303.55 | 1430.23 | 2584.81 | 300.84 | 301.58 | 236.32 | 1663.24 | 384.79 | 343.80 | 1060.91 | 266.23 | 877.43 |
|  | 7 | 2145.39 | 1681.60 | 3024.87 | 320.63 | 285.80 | 253.41 | 1957.15 | 430.69 | 350.87 | 1324.58 | 205.30 | 777.21 |
|  | 8 | 283.65 | 1538.58 | 4646.24 | 258.10 | 168.61 | 183.77 | 1396.15 | 461.83 | 308.64 | 1837.84 | 102.44 | 760.33 |
|  | 9 | 806.40 | 2127.62 | 5010.37 | 193.92 | 132.89 | 173.86 | 1693.29 | 254.36 | 405.84 |  |  |  |

5-HT: serotonin; 5-HIAA: 5-hydroxyindoleacetic acid; NE: noradrenaline. Data was lost in some samples.

**Table S5. Effects of CaCl_2_ microinjection in the DRN on neuronal activity in sleep-wake regulating nucleus (raw data).**

| **Group** | **No.** | **DRN**  **c-Fos (+) ratio in**  **serotonergic neurons (%)** | **VLPO**  **c-Fos (+) ratio in**  **GABAergic neurons (%)** | **Pef**  **c-Fos (+) ratio in**  **orexinergic neurons (%)** | **TMN**  **c-Fos (+) ratio in**  **histaminergic neurons (%)** | **LC**  **c-Fos (+) ratio in**  **noradrenergic neurons (%** |
| --- | --- | --- | --- | --- | --- | --- |
| **Vehicle** | 1 | 11.98757 | 37.12121 | 27.4531 | 24.91624 | 32.25806 |
|  | 2 | 21.19556 | 54.73934 | 0 | 20.51948 | 26.92308 |
|  | 3 | 14.58368 | 42.38281 | 19.81982 | 26.23338 | 23.91304 |
|  | 4 | 21.21231 | 47.77563 | 16.36364 |  | 30.62049 |
|  | 5 | 24.34282 | 50.31348 | 21.42857 | 19.72318 | 21.57407 |
|  | 6 | 27.77778 | 58.73786 | 23.07692 | 14.85714 | 25.9887 |
|  | 7 |  | 53.17073 |  |  | 21.95122 |
|  | 8 |  | 41.04803 |  |  |  |
|  | 9 |  | 48.58044 |  |  |  |
| **CaCl_2_ 25 nmol** | 1 | 39.27678 | 25.34247 | 56.06061 | 29.45597 | 44.96568 |
|  | 2 | 34.49138 | 34.60076 | 42.85714 | 28.80681 | 40.15573 |
|  | 3 | 42.30769 | 36.38498 | 39.47368 | 26.59558 | 45.27027 |
|  | 4 | 34.79951 | 29.79275 | 38.88889 | 22.42152 | 41.44088 |
|  | 5 | 29.45887 | 33.10658 | 34.42623 | 15.07538 | 31.96011 |
|  | 6 | 36.33695 | 25 | 42.85714 | 19.88304 | 42.98942 |
|  | 7 |  | 27.23005 |  |  | 33.33333 |
|  | 8 |  | 28.11736 |  |  |  |
|  | 9 |  | 31.50685 |  |  |  |
| **CaCl_2_ 50 nmol** | 1 | 26.9987 | 28.04054 | 62.5 | 20.52618 | 48.23232 |
|  | 2 | 40.34303 | 31.77966 | 50 | 21.46552 | 47.5 |
|  | 3 | 32.02171 | 30.52632 | 38.88889 | 28.38875 | 51.38889 |
|  | 4 | 25.42534 | 37.37024 | 40.87591 | 26.21359 | 42.10526 |
|  | 5 | 33.85409 | 31.44105 | 43.75 | 15.90909 | 46.34682 |
|  | 6 |  | 31.86813 | 41.09589 |  | 56.21212 |
|  | 7 |  | 31.88406 |  |  | 62.5 |
|  | 8 |  | 22.80702 |  |  |  |
|  | 9 |  | 33.46457 |  |  |  |

DRN: dorsal raphe nucleus; LC: locus coeruleus; NE: norepinephrine; Pef: perifornical nucleus; TMN: tuberomammillary nucleus; VLPO: ventrolateral preoptic nucleus. Data was lost or not detected in some samples.
